# Comparison of clinical outcome between incremental peritoneal dialysis and conventional peritoneal dialysis: a propensity score matching study

**DOI:** 10.1080/0886022X.2021.1960564

**Published:** 2021-08-15

**Authors:** Su Mi Lee, Yoon Sung Min, Young Ki Son, Seong Eun Kim, Won Suk An

**Affiliations:** Department of Internal Medicine, Dong-A University, Busan, Republic of Korea

**Keywords:** Peritoneal d, ialysis, survival, hospitalization, peritonitis, diabetes mellitus

## Abstract

**Background:**

Incremental peritoneal dialysis (iPD) can be useful in patients with residual renal function (RRF). RRF was well preserved and similar survival was shown in iPD compared to conventional PD (cPD) in previous study. However, the long-term survival of iPD remains unclear compared to cPD in diabetic patients. This study evaluated whether patient survival, hospitalization and peritonitis, and PD survival in iPD were lower than cPD or not.

**Methods:**

We conducted a 12-year retrospective observational study of 303 PD patients (232 cPD and 71 iPD) using propensity score matching by age, gender, and diabetes mellitus (DM). Finally, 78 cPD patients and 39 iPD patients were included and 44 patients had DM. Incremental PD was defined as starting PD with two or three manual exchanges per day.

**Results:**

The median duration of iPD was 24.1 months and iPD had higher RRF than cPD. Compared to cPD, the patient survival, PD survival and hospitalization benefits were not found in iPD but diabetic iPD patients had significantly longer survival and less hospitalization. Cumulative risk for peritonitis was lower iPD and PD duration of iPD was longer than those of cPD. The iPD was an independent factor associated with survival in patients with DM.

**Conclusions:**

Incremental PD may be a safe PD modality to initiate and maintain PD in less uremic patients with tolerable RRF. Incremental PD would be a benefit for survival in diabetic patients. Further prospective studies are necessary to confirm the effectiveness of iPD in PD patients with similar RRF.

## Introduction

End-stage renal disease (ESRD) is a global public health problem, and its frequency has steadily increased with time. According to the US Renal Data System (USRDS), the prevalence of ESRD reached over 720,000 cases in 2016 and over 270,000 cases had diabetes mellitus (DM) [1]. Among the diabetics, 17.1% had kidney transplantation (KT), 76.0% were started on hemodialysis (HD), and 6.9% on peritoneal dialysis (PD). The total number of patients with renal replacement therapy (RRT) in Korea has increased every year, exceeding 100,000 in 2018 and 48.8% had DM [2]. Although the prevalent number of patients with RRT continues to grow, the incidence of patients with PD has steadily declined since 2006. The risk of catheter-related complications, continuous exposure of peritoneal membrane from high glucose PD solution, and fear of having to do dialysis by themselves and the hassle of having to do it can lead to hesitation in PD [3–5].

Incremental peritoneal dialysis (iPD) was first introduced in the late 1990s, and iPD definition was unclear between earlier initiation and fewer dwell times at that time. IPD was defined as the frequencies of initiating PD exchanges less than four times a day, not earlier PD start [6]. In 2020, International Society for Peritoneal Dialysis (ISPD) recommended the definition of iPD as intention of increasing the PD prescription if and when RRF declines [7]. It may induce longer residual renal function (RRF) preservation, lower financial cost, or lesser time burden of PD treatment [8]. In addition, patients on iPD would have a chance of less peritoneal glucose exposure. However, there is still debate on whether PD should begin with a full dose with four exchanges per day or with incremental doses. Accordingly, in this study, we investigated the effect of iPD, with an emphasis on clinical outcomes including patient survival, PD survival, and peritonitis in patients starting PD for a long-term follow-up period. In addition, there are few comparative studies of the clinical outcomes of iPD and cPD based on the presence or absence of DM. Therefore, we evaluated clinical outcomes according to the presence of DM.

## Methods

### Study design and patients

This study analyzed retrospectively collected clinical data between January 2007 and December 2018 at the Dong-A University Hospital, Busan, Republic of Korea. A total of 303 PD patients (cPD (*n* = 232) vs. iPD (*n* = 71)), who were over 19 years of age were included. Patients who had started HD before the PD catheter insertion, had done PD with an automated cycler, or had a total duration of PD less than 6 months were excluded from the sample. All enrolled PD patients were cared by nephrologists within 3–6 months of starting dialysis. All patients had PD catheters implanted by experienced general surgeons using a midline or vertical incision under local anesthesia.

The patients were divided into two groups: iPD and conventional PD (cPD). Conventional PD was defined as the initiation of PD with four exchanges with 2 L per day, seven days a week, for continuous ambulatory peritoneal dialysis (CAPD). IPD was defined as starting PD with two or three manual exchanges per day [7]. IPD was started and maintained by physician’s decision, if the patients were tolerable to body edema or uremic symptoms. Therefore, relatively healthier patients can be chosen for iPD modality. We further restricted matching by age, gender, and the presence of DM. The final sample included 78 cPD patients and 39 iPD patients. Of the 44 patients with DM, 15 had iPD and 29 had cPD. PD solutions (Baxter Healthcare Corporation, Deerfield, IL, or Fresenius Medical Care, Bad Homburg, Germany) were used by physician’s decision according to status of patients’ edema.

This study was approved by the Dong-A University Institutional Review Board (DAUHIRB-19-193). Informed consent was waived because of the retrospective design of this study. The data including patient records and information were anonymized and de-identified prior to analysis. All clinical investigations were performed in accordance with the Declaration of Helsinki.

### Clinical outcomes

The primary endpoint was defined as the clinical outcomes, such as overall survival, PD survival, hospitalization, and peritonitis. Peritonitis was defined by the presence of the signs and symptoms of peritoneal inflammation and peritoneal effluent with a white blood cell count of >100 cells/mm^3^ and a polymorphonuclear leukocyte count of >50% [9,10]. Hospitalization was defined as an event requiring at least an admission to an in-patient unit during the investigation period. Causes of hospitalization included cardiovascular disease and cerebrovascular disease. Causes for drop-out were death, transfer to HD, or KT. Dialysis adequacy was assessed using weekly Kt/V (peritoneal + renal) and creatinine clearance. Anuria was defined as urine volume of <100 mL per day.

### Statistical analysis

Data were expressed as means ± SD for continuous variables and as frequencies (%) for categorical variables. Continuous variables were assessed using Student’s *t-*test, Mann–Whitney’s *U* test, and Wilcoxon’s rank sum test. Categorical variables were assessed using the chi-squared test. To identify peritonitis-free survival, technical survival, and patient survival in the two groups, the Kaplan–Meier analysis and log-rank tests were performed. Proportional hazard assumption was checked using graphical diagnostics based on the Schoenfeld residuals. The test is not statistically significant for iPD (*p* = .078). A multivariate Cox proportional hazards regression analysis to identify the effect of iPD on mortality was performed. The variables included age, gender, DM, serum creatinine (sCr), and the presence of iPD. All analyses were performed using SPSS software version 18.0 (IMB Corp., Armonk, NY), with a significance level of a *p* value of <.05.

## Results

### Patient characteristics

Baseline data are reported in Table 1. A total of 303 PD patients were enrolled in this study during the 12-year investigational period from 2007 to 2018. The mean age of the patients was 59.3 ± 14.0 years, and 59.4% of the participants were male. The proportion of patients with DM amounted to 42.2%. Before using propensity score matching, 232 patients were in the cPD group, and 71 patients were in the iPD group. The body mass index (kg/m^2^) was 23.7 ± 3.2 in cPD patients and 23.9 ± 2.9 iPD patients (*p* = .535). The body weight (kg) was 63.2 ± 11.3 in cPD patients and 62.2 ± 9.1 iPD patients (*p* = .607). The number of patients using diuretics is 64 (82.1%) in cPD and 31 (79.5%) in iPD. The number of patients waitlisted for KT is 36 (46.2%) in cPD and 18 (46.2%) in iPD. In the iPD group, sCr, and blood urea nitrogen (BUN) levels were lower than in those in the cPD group.

**Table 1.** Baseline characteristics of enrolled peritoneal dialysis patients.

Characteristics	Before propensity score matching			After propensity score matching		
	Conventional PD	Incremental PD	*p* Value	Conventional PD	Incremental PD	*p* Value
Patients, *n*	232	71		78	39	
Age (years)	58.8 ± 14.3	60.8 ± 12.6	.293	58.2 ± 13.8	58.2 ± 13.7	.989
Male, *n* (%)	143 (61.6)	39 (54.9)	.313	47 (60.3)	23 (59.0)	1.000
Diabetes mellitus, *n* (%)	93 (40.1)	35 (49.3)	.169	29 (37.2)	15 (38.5)	1.000
Duration of PD (month)	29.2 ± 22.1	31.2 ± 23.4	.502	39.5 ± 24.7	49.8 ± 26.7	.041
Laboratory findings						
Creatinine (mg/dL)	8.9 ± 3.4	7.2 ± 2.7	<.001	10.1 ± 3.9	7.3 ± 2.7	<.001
GFR (mL/min/1.73 m^2^)	6.8 ± 4.6	8.0 ± 3.4	.052	5.9 ± 3.4	8.3 ± 3.8	.001
BUN (mg/dL)	93.8 ± 30.0	79.5 ± 25.7	.001	98.0 ± 32.3	79.7 ± 27.3	.003
Albumin(g/dL)	3.5 ± 0.5	3.5 ± 0.5	.888	3.5 ± 0.5	3.5 ± 0.4	.673
Potassium (mmol/L)	4.6 ± 0.8	4.6 ± 0.7	.672	4.6 ± 0.8	4.5 ± 0.7	.439
Calcium (mg/dL)	7.8 ± 1.0	7.8 ± 1.0	.744	7.8 ± 1.1	7.9 ± 1.0	.531
Phosphorus (mg/dL)	5.4 ± 1.7	5.2 ± 1.2	.257	5.7 ± 1.9	5.0 ± 1.1	.010
Hemoglobin (g/dL)	9.2 ± 1.4	9.6 ± 1.1	.081	9.1 ± 1.4	9.6 ± 1.0	.069
HbA1c (%)	7.0 ± 1.7	7.3 ± 1.9	.285	6.9 ± 1.3	6.9 ± 1.9	.931
Urine volume (mL/day)	645.4 ± 383.5	718.9 ± 353.3	.527	597.4 ± 441.7	748.6 ± 395.8	.378

PD: peritoneal dialysis; GFR: glomerular filtration rate; BUN: blood urea nitrogen; HbA1c: glycosylated hemoglobin; PD: peritonea dialysis.

Data are expressed as means ± SD or frequency.

The mean age, gender, and presence of DM were not significantly different between the two groups. The levels of BUN and sCr were significantly lower, and glomerular filtration rate (GFR) was significantly higher in the iPD group than in the cPD group. There were no significant differences in albumin, potassium, calcium, hemoglobin, and glycated hemoglobin (HbA1c) levels between the two groups. Mean PD duration of iPD patients was longer than that of cPD patients.

Baseline data in patients with DM are reported in Table 2. Among diabetic patients, 15 had iPD and 29 had cPD. The sCr levels were significantly lower and mean PD duration was significantly longer in the iPD group than in the cPD group. There were no significant differences in GFR, BUN, albumin, potassium, calcium, phosphorus, hemoglobin, and HbA1c levels between two groups. The score of the Charlson comorbidity index (CCI) is 7.6 ± 1.6 in DM patients. The score of CCI is 7.9 ± 1.7 in cPD and 7.2 ± 1.3 in iPD with DM (*p*=.188).

**Table 2.** Baseline characteristics of peritoneal dialysis patients with absence or presence of diabetes mellitus.

Characteristics	Absence of DM			Presence of DM		
	Conventional PD	Incremental PD	*p* Value	Conventional PD	Incremental PD	*p* Value
Patients, *n*	49	24		29	15	
Age (years)	55.2 ± 15.3	54.7 ± 15.2	.888	63.2 ± 9.1	63.7 ± 8.7	.864
Male, *n* (%)	25 (51.0)	12 (50.0)	1.000	22 (75.9)	11 (73.3)	1.000
Duration of PD (month)	45.0 ± 27.9	49.7 ± 31.0	.517	30.1 ± 14.4	49.9 ± 18.7	<.001
Laboratory findings						
Creatinine (mg/dL)	11.3 ± 4.0	8.0 ± 3.0	.001	8.1 ± 2.7	6.1 ± 1.7	.016
GFR (mL/min/1.73 m^2^)	4.8 ± 2.4	7.5 ± 4.2	.008	7.6 ± 4.2	9.5 ± 2.7	.110
BUN (mg/dL)	104.4 ± 34.1	81.8 ± 30.2	.007	87.1 ± 26.0	76.2 ± 22.4	.177
Albumin(g/dL)	3.5 ± 0.5	3.6 ± 0.4	.951	3.4 ± 0.5	3.5 ± 0.5	.540
Potassium (mmol/L)	4.5 ± 0.7	4.6 ± 0.7	.722	4.8 ± 0.9	4.3 ± 0.7	.114
Calcium (mg/dL)	7.8 ± 1.2	7.8 ± 1.0	.915	7.7 ± 0.8	8.1 ± 1.0	.163
Phosphorus (mg/dL)	6.1 ± 2.1	5.0 ± 1.1	.004	5.0 ± 1.2	4.9 ± 1.2	.773
Hemoglobin (g/dL)	9.1 ± 1.4	9.3 ± 0.8	.341	9.2 ± 1.3	10.0 ± 1.2	.060
HbA1c (%)				7.7 ± 1.1	8.1 ± 1.8	.374

PD: peritoneal dialysis; GFR: glomerular filtration rate; BUN: blood urea nitrogen; HbA1c: glycosylated hemoglobin; PD: peritonea dialysis.

Data are expressed as means ± SD or frequency.

### Patient and PD survival

**Figure 1.**
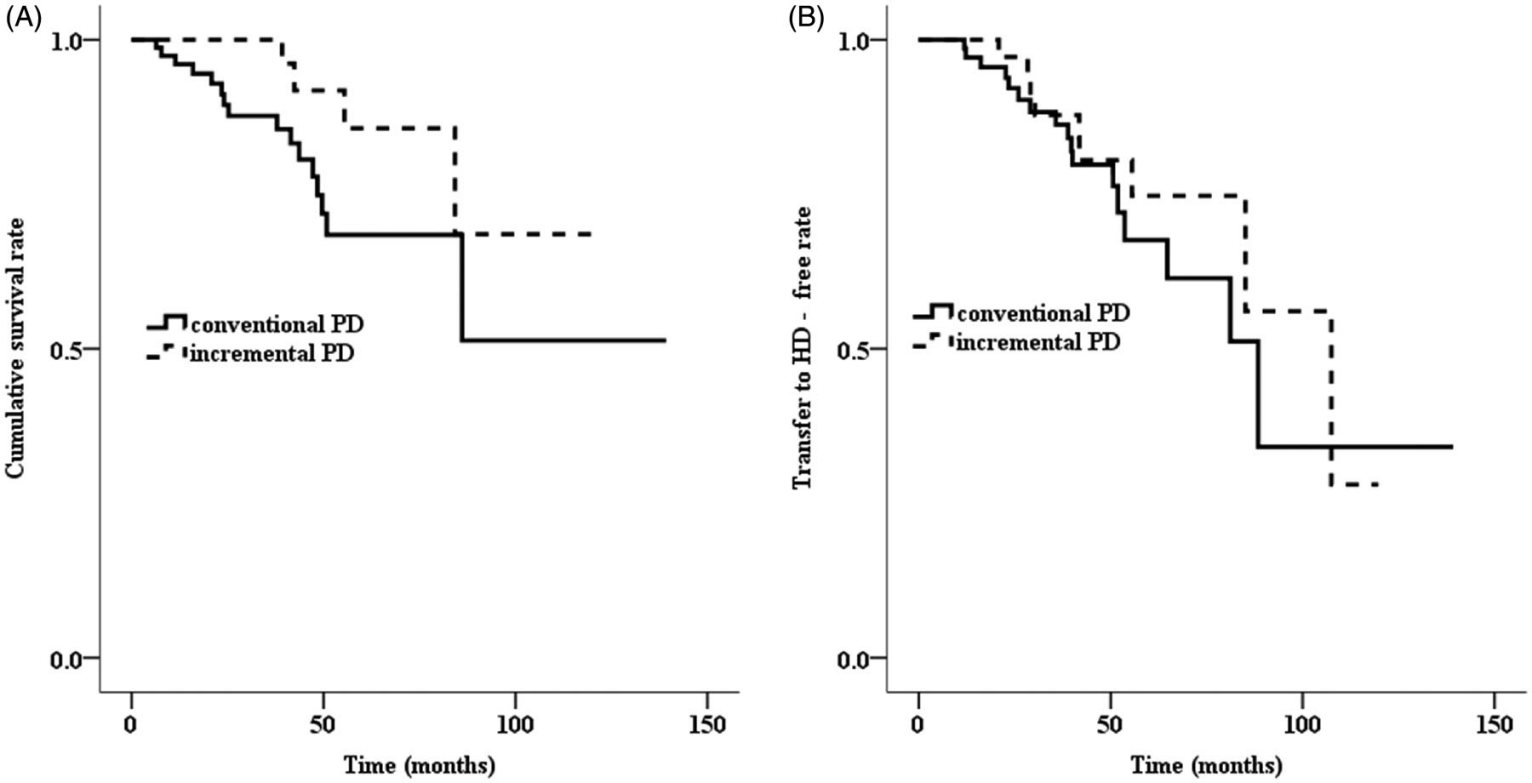
The Kaplan–Meier curve for (A) death and (B) transfer to HD (log-rank test, *p*= .067, *p*= .409).

**Figure 2.**
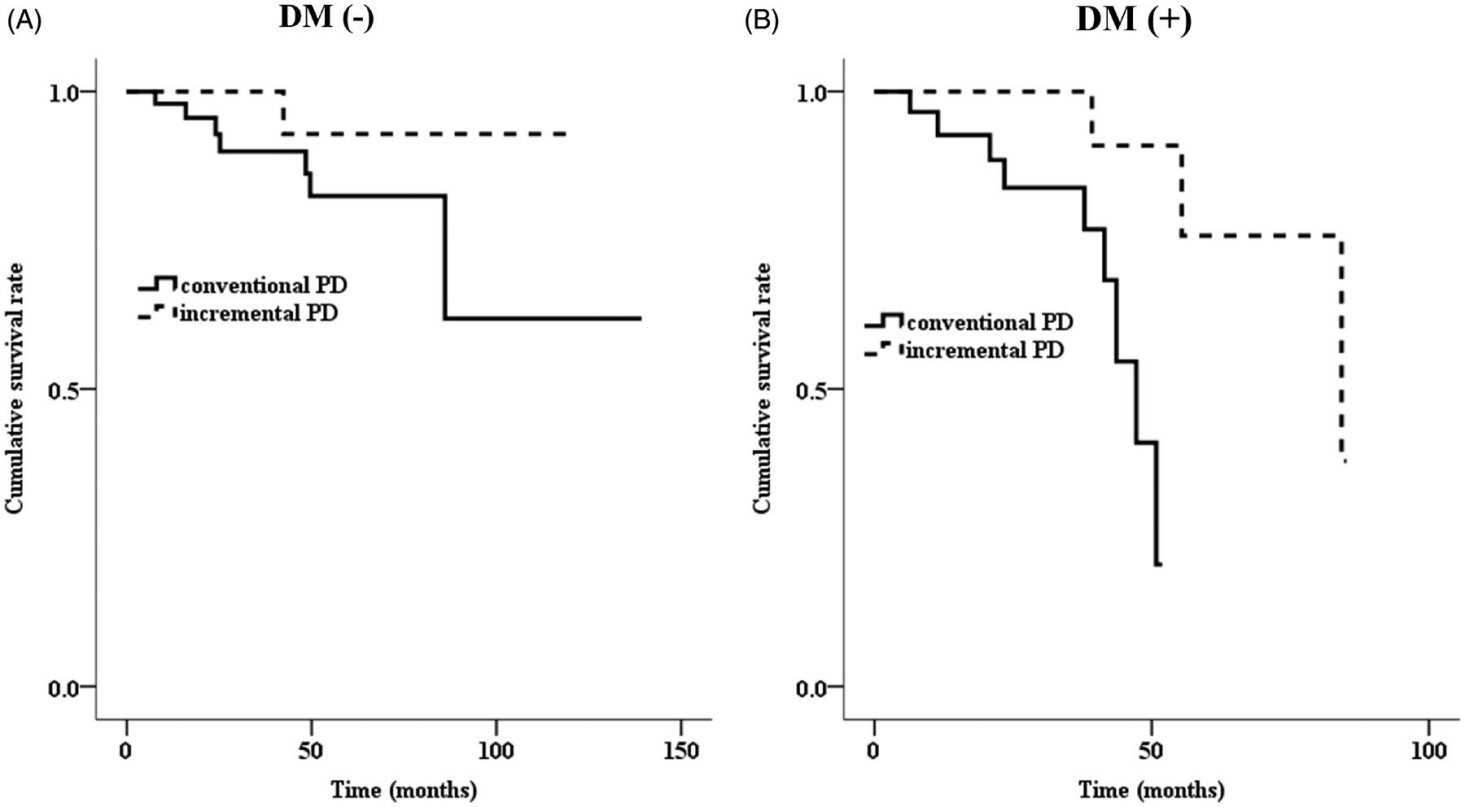
The Kaplan–Meier curve for death according to (A) absence or (B) presence of DM (log-rank test, *p*= .180, *p*= .009).

The median duration of iPD was 24.1 months (15.4–36.8). Incremental PD patients quitted PD due to death (10.3%), KT (7.7%), or transfer to HD (23.1%). In the iPD group, two patients died due to cerebral infarction, one due to ischemic heart disease, and one due to superior mesenteric artery thromboembolism. Conversely, three patients died due to cerebral infarction, four due to cerebral hemorrhage, one due to ischemic heart disease, one due to hepatic encephalopathy, and eight due to infection in the cPD group. The cumulative risk for mortality between iPD and cPD patients was not significantly different (log-rank test, *p* = .067, Figure 1(A)). However, survival benefit was more prominent in the patients with iPD than in those with cPD among the patients with DM (log-rank test, *p* = .009, Figure 2(B)). The independent effect of iPD on mortality was not significant using multivariate Cox proportional hazard models in 117 PD patients (Table 3). The iPD remained as an independent variable associated with mortality in PD patients with DM (Table 4).

**Table 3.** Multivariate Cox proportional model for mortality among 117 enrolled PD patients.

Characteristics	Univariate		Multivariate	
	HR (95% CI)	*p* Value	HR^a^ (95% CI)	*p* Value
Age (years)	1.065 (1.030–1.101)	<.001	1.060 (1.023–1.099)	.001
Male, *n* (%)	1.285 (0.653–2.527)	.468	1.369 (0.684–2.741)	.375
Diabetes mellitus, *n* (%)	2.030 (1.013–4.067)	.046	1.621 (0.764–3.441)	.208
Creatinine (mg/dL)	0.873 (0.781–0.977)	.018	0.950 (0.838–1.077)	.422
Incremental peritoneal dialysis, *n* (%)	0.645 (0.308–1.351)	.245	0.504 (0.230–1.104)	.087

^a^Clinical parameters (age, gender, diabetes mellitus, and creatinine) were examined with incremental peritoneal dialysis.

**Table 4.** Cox proportional model for mortality in 44 patients with diabetes mellitus.

Characteristics	Univariate	
	HR (95% CI)	*p* Value
Age (years)	1.061 (0.988–1.140)	.104
Male, *n* (%)	1.201 (0.411–3.505)	.738
Creatinine (mg/dL)	0.983 (0.796–1.214)	.872
Incremental peritoneal dialysis, *n* (%)	0.148 (0.032–0.678)	.014

The cumulative risk for mortality between DM and non-DM was not significantly different in the patients with iPD (log-rank test, *p* = .910). Among the patients receiving cPD, cumulative risk for mortality was lower in the non-DM group than in the DM group (log-rank test, *p* = .005).

The incidence of transfer to HD was nine (23.1%) patients in the iPD group and 17 (21.8%) patients in the cPD group. Among the patients on iPD, the causes of transition were a recurrent peritonitis in six patients, one due to inadequate dialysis, one due to colitis, and one due to ovarian cystic tumor. The cumulative risk for HD transition was similar between iPD and cPD patients (log-rank test, *p* = .409, Figure 1(B)). However, PD durations of iPD were statistically longer than those of cPD. Among the patients with DM, cumulative risk for HD transition was similar between iPD group and cPD group (log-rank test, *p* = .112). The cumulative risk for HD transition in the non-diabetic patients was also similar between two groups (log-rank test, *p* = .794).

### Peritonitis and hospitalization

**Figure 3.**
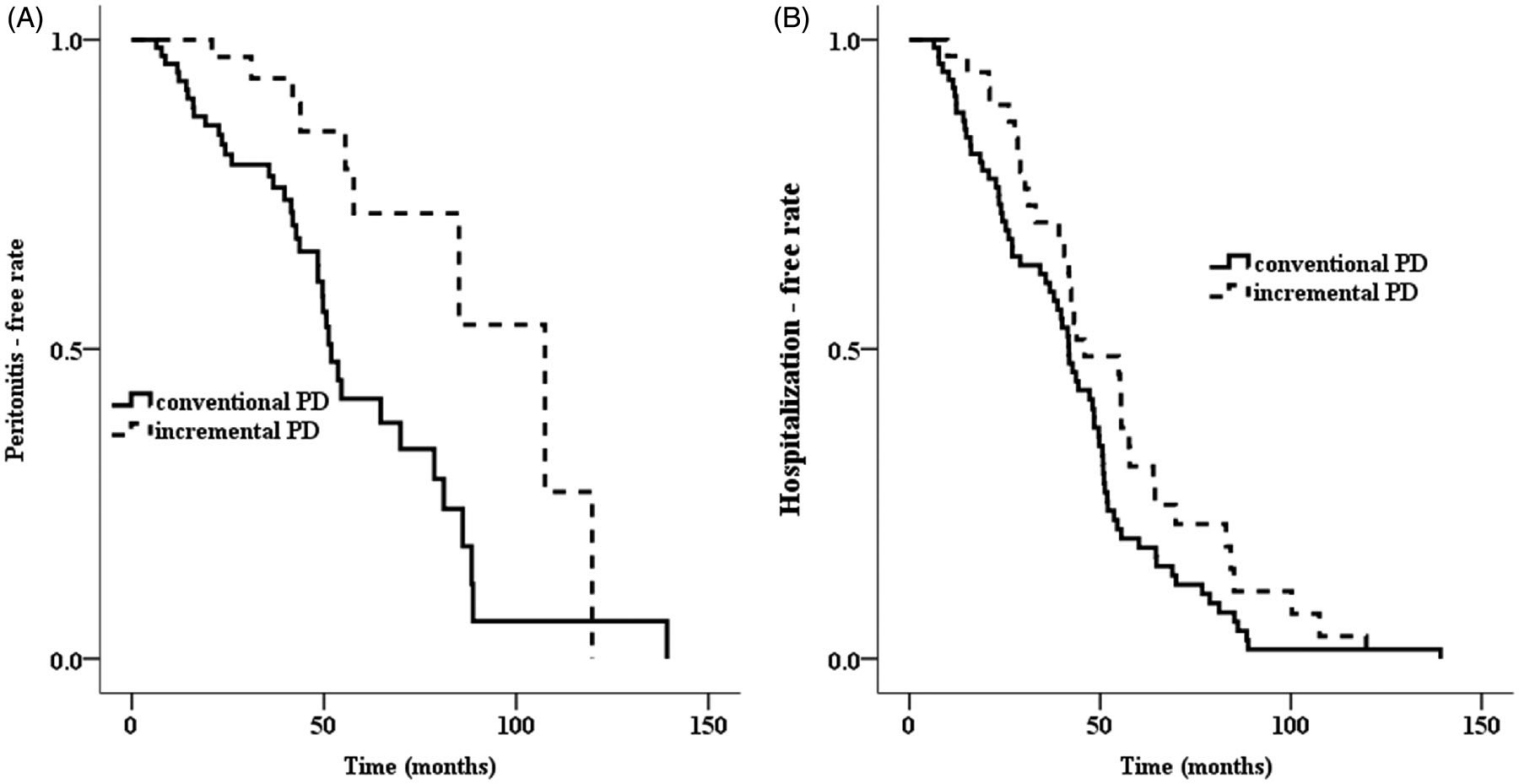
The Kaplan–Meier curve for (A) peritonitis and (B) hospitalization (log-rank test, *p*= .002, *p*= .058).

During a total follow-up of 413 patient-year, 47 episodes of peritonitis occurred, with an incidence of 1/9 patient-year. The incidence of peritonitis was 1/18 patient-year in the iPD patients and 1/7 patient-year in the cPD patients. The cumulative risk for peritonitis was lower in the iPD patients than in the cPD patients (*p* = .002, log-rank test, Figure 3(A)). Among the patients with DM, incidence of peritonitis was lower in the iPD group than in the cPD group (log-rank test, *p* = .001). The incidence of peritonitis in the non-diabetic patients was also lower in the iPD group than in the cPD group (log-rank test, *p* = .032).

The incidence of hospitalization was 1/5 patient-year in iPD patients and 1/4 patient-year in cPD patients. The cumulative rate for being hospitalization was lower in the iPD patients than in the cPD patients (*p* = .058, log-rank test, Figure 3(B)). Among diabetic patients, hospitalization rate was much lower in patients with iPD than in those with cPD (*p* = .003, log-rank test). Among the patients with DM, cumulative risk for being hospitalization was lower in the iPD group than in the cPD group (log-rank test, *p* = .003). The cumulative rate for being hospitalization in the non-diabetic patients was similar between two groups (log-rank test, *p* = .235).

## Discussion

In this study, we found that iPD strategy was not harm in terms of hospitalization, PD survival and mortality compared to cPD. In addition, iPD strategy in patients with DM was beneficial in terms of hospitalization and mortality compared to starting with conventional full dose PD. The iPD strategy, not meaning early PD starting, is now increasing. The impact of iPD on improving patient experience is apparent considering beneficial points of iPD such as quality of life, working activity, and degree of rehabilitation [11,12]. Sandrini et al. showed that patients with iPD had similar survival rates, lower hospitalization, a trend toward lower peritonitis incidence and slower reduction of renal function compared to cPD [13]. Lee et al. reported that iPD was beneficial for preserving RRF, even with similar technique for survival and mortality rates in comparison with cPD [14]. Still, there are no data about demonstrating the survival benefits caused by iPD in patients with DM. Based on our results, we suggest that iPD may improve not only patient survival but also reduce hospitalization compared to cPD in patients with DM. Although baseline sCr levels of the iPD patients with DM were better than that of cPD patients, Cox proportional hazard models also showed better survival in patients treated with iPD strategy. Therefore, we recommend iPD in patients starting PD if the CKD patients with DM are under less uremic condition with RRF.

A possible mechanism of iPD benefit may be less exposure of the glucose PD solution caused by fewer exchanging PD solutions. Numerous studies have compared iPD and cPD [13–16], but there were no reports that would compare iPD and cPD in a diabetic population treated with PD. The results of this study showed that clinical benefits of iPD were prominent in diabetic patients. Although iPD patients had better RRF than that of cPD patients, starting with iPD resulted in longer PD treatment periods even in diabetic PD patients. Our center has tried to incrementally increase the amounts of PD solution in iPD patients. When iPD started twice a day, PD solution was exchanged every 6 h and 12 h was maintained at an empty. Icodextrin solution was initially used for body edema control when iPD started thrice a day. Therefore, fewer exchanges and lower amounts of PD solutions were definitely related with less exposure of glucose PD solutions. Previous studies also showed the clinical benefit and experimental prevention of peritoneal mesothelial cell fibrosis caused by less exposure or lower glucose solutions [17,18]. Beneficial effects of iPD patients with DM may be related with fewer glucose exposures.

In this study, the iPD group showed a lower peritonitis incidence and a longer PD duration, and this is an important advantage for the PD patients, because one major cause of drop-out from PD is a high rate of peritonitis. The lower number of exchange and the longer duration of dry status are associated with a lower peritonitis incidence [19,20]. It also minimizes the gradual accumulation of membrane damage to delay the time of ultrafiltration failure, if the patient complies with proper salt and water intake. Little exposure of dialysate makes little metabolic complications such as hyperglycemia and protein loss into the dialysate [21,22]. Hyperglycemia is caused by using dialysate containing high concentrations of glucose and is thought to be a contributing factor for vascular problems, such as stroke and coronary artery disease. This is another reason the iPD group with DM showed a lower mortality in this study.

Although the exact pathophysiologic mechanism of iPD benefit remained unclear, preserving RRF was suggested as one of the important clues in recent studies [12,14]. This study also showed relatively preserved urine volumes after 1 year in the iPD group (from 690.7 ± 375.6 mL/day to 593.3 ± 547.1 mL/day) compared to the cPD group (from 658.3 ± 462.8 mL/day to 423.0 ± 533.8 mL/day), although the difference was not statistically significant (Supplementary Table 1). Given that preserving RRF is associated to lower morbidity and mortality in the PD patients, this point can present a clinical evidence for supporting that iPD strategy shows at least as good as survival rate compared to cPD strategy [15]. Preserved RRF may also affect the longer median iPD duration over 2 years in this study.

In the past, when the exact concept of iPD was not established, iPD was defined as PD modality with two or fewer dialysate exchanges [13]. In a different study, three or fewer exchanges were defined as iPD with the monitoring of GFR and Kt/V [15]. The ISPD defines iPD as less PD prescription in patient with RRF. Therefore, physician can increase the PD prescription if and when RRF declines [7]. We began iPD by two or three times of PD dialysate exchange with a maximum of 6 L of PD solution a day and iPD was selected if the patient had little signs of edema or uremia. The amount of PD solution is also important. We incrementally increased one exchange PD solution volume from 1.0 L to 1.5 L within 6 months or from 1.5 L to 2.0 L within 1 year. At the beginning of iPD, large amounts of PD solution can cause abdominal discomfort and decreased food intake, and increased dialysis dose can cause increased protein loss. Notably, dialysate exchanges and PD solution volumes should be increased according to body edema and uremic status.

This study has several limitations. First, this was a retrospective study, and we did not periodically measure RRF in the whole PD group. Therefore, much data about RRF during the study were omitted, and the size of dataset was too small to get statistical significance about iPD’s benefit for preserving RRF. Second, the participants of this study were from a single center. The results of the study could be affected by the region. Third, starting iPD and transition from iPD to full PD was decided by patients tolerability if just dependent on physician’ decision. In spite of these limitations, this study support that starting with iPD is definitely not harmful even in diabetic PD patients with tolerable RRF.

In conclusion, iPD may be a safe PD modality to initiate and maintain PD in less uremic patients with tolerable RRF. In addition, iPD would be a benefit for patient survival, particularly in diabetic PD patients. Further prospective studies are necessary to confirm the effectiveness of iPD in PD patients with similar RRF.

## Supplementary Material

Supplemental Material

## Abbreviations

BUN: blood urea nitrogen
CAPD: continuous ambulatory peritoneal dialysis
CCI: Charlson comorbidity index
cPD: conventional peritoneal dialysis
DM: diabetes mellitus
ESRD: end-stage renal disease
GFR: glomerular filtration rate
HbA1c: glycated hemoglobin
HD: hemodialysis
iPD: incremental peritoneal dialysis
ISPD: International Society for Peritoneal Dialysis
KT: kidney transplantation
PD: peritoneal dialysis
PD: peritoneal dialysis
PD: peritoneal dialysis
PD: peritoneal dialysis
RRF: residual renal function
RRT: renal replacement therapy
sCr: serum creatinine
USRDS: United States Renal Data System
