# Supplementary material for: Comparison of clinical outcome between incremental peritoneal dialysis and conventional peritoneal dialysis: a propensity score matching study
Source: Ren Fail. 2021 Aug 15;43(1):1222–8. doi: 10.1080/0886022X.2021.1960564 (PMC8381909; doi:10.1080/0886022X.2021.1960564)
Supplement: Supplemental Material [file IRNF_A_1960564_SM2133.pdf]

SUPPLEMENTARY TABLE 1

Changes of dialysis adequacy

Characteristics	Baseline			After 1year		
	Conventional PD	Incremental PD	*p* value	Conventional PD	Incremental PD	*p* value
Patients, n	20	9		20	9	
Total weekly Kt/V	2.5 ± 1.3	2.1 ± 0.7	0.311	2.0 ± 0.6[a]	1.8 ± 0.3	0.925
Weekly peritoneal Kt/V	1.6 ± 0.6	1.1 ± 0.3	0.014	1.5 ± 0.3	1.5 ± 0.4[a]	0.863
Weekly renal Kt/V	0.9 ± 1.1	0.9 ± 0.5	0.665	0.5 ± 0.7[a]	0.3 ± 0.4[a]	1.000
Total CCr (L/week/1.73m^2)	108.0 ± 78.6	84.3 ± 31.5	0.480	65.3 ± 27.4[a]	71.8 ± 32.3	0.540
Peritoneal CCr (L/week/1.73m^2)	45.0 ± 21.6	33.9 ± 12.1	0.265	41.0 ± 12.7	42.7 ± 12.3	0.750
Renal CCr (L/week/1.73m^2)	47.8 ± 41.4	43.7 ± 25.5	1.000	24.0 ±3 2.7[a]	20.4 ± 24.7	0.978
Urine volume (mL/day)	658.3 ± 462.8	690.7 ± 375.6	0.741	423.0 ± 533.8	593.3 ± 547.1	0.400

Data are expressed as means ± SD.
p value for nonparametric Mann-Whitney *U* test comparing data between patients with conventional PD and those with incremental PD.
The nonparametric Wilcoxon exact rank sum test was used to compare baseline data with after 1years data.
[a]*p* value <0.05 (mean values are significantly different from baseline data).
Abbreviations: CCr, creatinine clearance; PD, peritoneal dialysis
